# Congenital cytomegalovirus, parvovirus and enterovirus infection in Mozambican newborns at birth: A cross-sectional survey

**DOI:** 10.1371/journal.pone.0194186

**Published:** 2018-03-14

**Authors:** Lola Madrid, Rosauro Varo, Sonia Maculuve, Tacilta Nhampossa, Carmen Muñoz-Almagro, Enrique J. Calderón, Cristina Esteva, Carla Carrilho, Mamudo Ismail, Begoña Vieites, Vicente Friaza, María del Carmen Lozano-Dominguez, Clara Menéndez, Quique Bassat

**Affiliations:** 1 Centro de Investigação em Saúde de Manhiça (CISM), Maputo, Mozambique; 2 ISGlobal, Barcelona Ctr. Int. Health Res. (CRESIB), Hospital Clínic - Universitat de Barcelona, Barcelona, Spain; 3 Molecular Microbiology Department, Institut de Recerca Sant Joan de Déu, University Hospital Sant Joan de Déu, Barcelona, Spain; 4 CIBER de Epidemiología y Salud Pública CIBERESP, Instituto de Salud Carlos III, Madrid, Spain; 5 School of Medicine. Universitat Internacional de Catalunya, Barcelona, Spain; 6 Instituto de Biomedicina de Sevilla, Hospital Universitario Virgen del Rocío/CSIC/Universidad de Sevilla, Seville, Spain; 7 Department of Pathology, Maputo Central Hospital, Maputo, Mozambique; 8 Faculty of Medicine, Eduardo Mondlane University, Maputo, Mozambique; 9 Department of Microbiology, Hospital Universitario Virgen del Rocío, Seville, Spain; 10 Department of Pathology, Hospital Universitario Virgen del Rocío, Seville, Spain; 11 ICREA, Barcelona, Spain; 12 Pediatric Infectious Diseases Unit, Pediatrics Department, Hospital Sant Joan de Déu (University of Barcelona), Barcelona, Spain; University of British Columbia, CANADA

## Abstract

**Background:**

Congenital cytomegalovirus (cCMV) infection is the most prevalent congenital infection acquired worldwide, with higher incidence in developing countries and among HIV-exposed children. Less is known regarding vertical transmission of parvovirus B19 (B19V) and enterovirus (EV). We aimed to assess the prevalence of CMV, B19V and EV vertical transmission and compare results of screening of congenital CMV obtained from two different specimens in a semirural Mozambican maternity.

**Methods:**

A cross sectional study was conducted among pregnant mothers attending Manhiça District Hospital upon delivery. Information on maternal risk factors was ascertained. Dried umbilical cord (DUC) samples were collected in filter paper for CMV, B19V and EV detection by real-time polymerase chain reaction (RT-PCR), and nasopharyngeal aspirates (NPA) to test for CMV by RT-PCR. Maternal blood samples and placental biopsy samples were also obtained to investigate CMV maternal serology, HIV status and immunopathology.

**Results:**

From September 2014 to January 2015, 118 mothers/newborn pairs were recruited. Prevalence of maternal HIV infection was 31.4% (37/118). CMV RT-PCR was positive in 3/115 (2.6%) of DUC samples and in 3/96 (6.3%) of NPA samples obtained from neonates. The concordance of the RT-PCR assay through DUC with their correspondent NPA sample was moderate (Kappa = 0.42 and p<0.001. No differences on cCMV prevalence were found among HIV-exposed and unexposed. All (100%) mothers were seropositive for CMV IgG. RT-PCR of EV and B19V in DUC were both negative in all screened cases. No histological specific findings were found in placental tissues. No risk factors associated to vertical transmission of these viral infections were found.

**Conclusions:**

This study indicates the significant occurrence of vertical transmission of CMV in southern Mozambique. Larger studies are needed to evaluate the true burden, clinical relevance and consequences of congenital infections with such pathogens in resource-constrained settings.

## Background

Despite the impressive reduction in child mortality in the last decades, neonatal mortality has declined more slowly and now accounts for nearly half (45%) of all under-5 child deaths[1]. Congenital and perinatal infections are well-known causes of neonatal morbidity and mortality and stillbirths in high-income countries (HIC)[2–4]. However, estimates of the global burden of congenital infections and attributable stillbirths, neonatal disease, disability or deaths due to mother-to-child transmission (MTCT) of these infections in low-income countries (LIC) are limited on account of a generalized scarcity of data[5]. Despite the positive impact in terms of health outcomes shown by the introduction of screening and treatment policies for several pregnancy-relevant infections such as human immunodeficiency virus (HIV), syphilis or malaria[6, 7]; pathogens that may also be vertically transmitted beyond these infections are rarely the focus of clinical practice and research[8, 9].

Several viruses, including among others cytomegalovirus (CMV), parvovirus B19V (B19V) and enterovirus (EV), may cause mild and self-limiting clinical manifestations among infected pregnant women, but more severe or even life-threatening disease in their offsprings. The Zika virus epidemic of 2016 in Latin America has contributed to highlight the emerging threat that maternal viral infections may carry for the health of the foetus and newborn[10].

Despite being the most prevalent congenital infection worldwide, congenital CMV infection (cCMV) remains largely neglected in the developed and developing world[11]. Although the global prevalence of cCMV has been reported to vary from approximately 0.2% to 2% (mean 0.65%), most of these studies have been conducted in high-income regions of Europe, USA or Japan were prevalence of cCMV ranges between 0.6–0.7%[12–14]. Data from LIC varies substantially, with some estimates peaking at 6–14%[15–17]. Higher overall rates of cCMV are found in countries with higher maternal CMV seroprevalence[12, 13, 18] and among infants exposed to HIV during pregnancy. Indeed, maternal HIV infection is thought to significantly increase (from 2.3% to 10.3%) the prevalence of cCMV in those HIV-exposed infants compared to those born to HIV-negative mothers, both in industrialized countries and also in LIC[19–23]. Furthermore, CMV infection seems to play a role as a cofactor for HIV disease progression in HIV/CMV co-infected newborns[24].

Different methods have been evaluated for cCMV screening based on saliva, urine and blood specimens, being saliva and urine the generally considered most appropriate samples[25]. Virus isolation from saliva or urine in rapid culture has been traditionally considered the standard method for identification of infants with cCMV but such methods appear unfeasible to perform for large screening efforts [26]. In contrast, real-time polymerase chain reaction (RT-PCR), also considered as gold standard [26–28], allows large numbers of specimens to be screened at a relatively low cost. However, RT-PCR based methods applied to dried-blood-spot (DBS), tested in countries where DBS are routinely collected for newborn metabolic screening, have shown a low sensitivity as a screening methodology[26]. Dried umbilical cord (DUC) sample-based PCR assays have demonstrated utility for diagnosis of cCMV, as part of retrospective investigations of the underlying aetiology of hearing impairment[29, 30]. Other samples such as nasopharyngeal aspirates (NPA) which are easily obtained and simple to store have been used to investigate respiratory pathogens of public health importance[31, 32]. However, to our knowledge, NPAs have not been evaluated as a screening methodology for cCMV diagnosis, although the Child Health and Mortality Prevention Surveillance Network (CHAMPS) aiming to know cause of death through innovative techniques such as the minimally invasive tissue sampling (MITS), proposes NPA as a standard specimen for diagnosis of cCMV[33, 34].

Knowledge gaps regarding the burden of other congenital infections associated to fatal foetal outcomes, such as B19V and EV, remain significant. B19V can cause a variety of foetal complications including spontaneous abortion, non-immune *hydrops foetalis* or intrauterine foetal death[35]. The epidemiology of B19V infection in pregnancy has been well studied in industrialized countries whereby prevalence has been estimated to vary from 1 to 5% in pregnant women with transmission rates to the foetus ranging between 17–33%[36]. However, the burden of this infection during pregnancy in LIC has been rarely documented and studies investigating in these settings active B19V infection in newborns have not been conducted.

Enteroviruses, which include coxsackieviruses and echoviruses, cause about one billion infections every year worldwide but their consequences during pregnancy have been seldom described[37]. Transplacental transmission of EV has been associated to stillbirths, non-immune *hydrops foetalis* and also severe neonatal infections, although the epidemiology and characterization of neonatal outcomes is not well documented[37].

Although data in LIC remain insufficient, higher burden of congenital infections may be assumed in regions like Southern Mozambique where HIV is highly prevalent and effective vaccines against pathogens such as rubella are partially or even not implemented[15, 38–41].

This is a pilot study exploring congenital acquisition of CMV, B19V and EV determined at birth. We additionally aimed to compare the results of two simple screening methodologies using RT-PCR for cCMV investigation, using DUC and NPA specimens obtained from the newborns.

## Methods

### Study site

The study was conducted in Manhiça, a semi-rural site in Southern Mozambique. The Manhiça Health Research Centre (CISM) runs a Demographic Surveillance System (DSS) in the area linked to a Hospital Morbidity Surveillance System (HMSS) ongoing at the Manhiça District Hospital (MDH) including all admitted children. A detailed description of MDH, CISM and the study area can be found elsewhere[42]. MDH is the referral hospital for the Manhiça district, covering a population of *circa* 183,000 inhabitants. The MDH includes adult and paediatric wards, together with a maternity, where an average of 3500–4000 deliveries takes place annually. Around 85% of all deliveries are institutionalized (A. Nhacolo, personal communication). It also includes an outpatient department and an antenatal care (ANC) clinic where pregnant women are routinely followed. As part of the National policy, all pregnant women are invited to attend ANC clinic during their pregnancy, where HIV testing and syphilis screening are routinely offered. Malaria transmission of moderate intensity is perennial with some seasonality and, intermittent preventive treatment during pregnancy (IPTp) for malaria prevention is recommended[42]. HIV prevalence in Manhiça district is amongst the highest in the world, with rates estimated at around 29% at the ANC clinic [40]. In 2013, MDH introduced WHO-recommended Option B+ for the prevention of mother-to-child HIV transmission[43], which is offered to mothers free of charge. No proactive strategies to screen for risk factors of neonatal sepsis or to prevent it are currently implemented in Mozambique.

### Study design and population

This observational pilot study was conducted at the delivery wards of MDH, between September 15^th^ 2014 and January 15^th^ 2015, running continuously during working hours (8:00–16:00) and working days. We recruited pregnant women upon delivery (regardless of gestational age (GA) and their offspring. Participants were eligible for inclusion if they were >18 years old and able and willing to participate in the study and to provide informed consent after an explanation of the study. As this was a pilot study, in order to obtain a more representative sample of the study population, only the first three women seen every day were approached for recruitment.

### Definitions

Gestational age was defined using fundal height, measured from the top of the mother's uterus to the top of the mother's pubic symphysis and assessed by a nurse specialist in maternal child health. A preterm baby was defined as that with a gestational age at birth of <37 weeks and a stillbirth case as an intrauterine death occurring after 28 weeks of GA. Low-birth weight was defined as weight at birth <2.500 grams[44]. Microcephaly was defined following the WHO growth standards[45]. Congenital CMV, B19V or EV infections were defined as detection of viral DNA/RNA by RT-PCR in dried cord umbilical samples obtained from neonates at birth[33]. Although cCMV diagnosis through NPA has not been validated, we also explored prevalence of CMV through positive RT-PCR in NPA specimens. Positive HIV status was defined according to national guidelines, which required for mothers two positive rapid testing (an initial discriminatory diagnostic test (Determine^®^) and a confirmatory test(Unigold^®^); and for children ≤ 18 months of age a positive rapid test in addition to a confirmatory positive PCR test.

### Study procedures

A placental biopsy and two drops of umbilical cord blood collected in filter paper (Whatman 903^®^, Florham Park, NJ) were obtained immediately after delivery in order to determine DNA/RNA of CMV, B19V and EV and assess placental histopathology. In addition, a NPA was collected from neonates within the first two hours from birth, using a bulb aspiration kit conveniently pre-filled with sterile saline (M-PRO NPAK nasopharyngeal aspiration kit^®^). At least 1ml of NPA specimen mixed with sterile saline was collected and stored with the objective of screening for CMV DNA. A blood sample was collected from mothers for assessing anti-CMV antibodies. Maternal HIV status was determined and recorded if not previously registered in antenatal source documents. Other screening test results routinely performed at the ANC clinic, such as syphilis screening (using Rapid Plasma Reagin) or haemoglobin determination were also recorded.

#### DNA extraction and real time -PCR for viral amplification

CMV and B19V DNA and EV RNA were extracted from 1 drop of DUC (around 50 ul) and CMV from 400 ul of NPA specimen by using the NucliSENS^®^ easyMag^®^ (bioMérieux, Marcy l’Etoile, France) instrument according to the manufacturer’s procedure and eluted in 25 μl of elution buffer for DUC and 50 ul for NPA. Samples were tested for the presence of CMV DNA, B19V DNA and EV RNA with three different real-time PCR, CMV Q- PCR alert Kit^®^, Elitech Group Molecular Diagnostics, RealStar^®^ Parvovirus B19V kit^®^, Altona Diagnostics and and a published in-house real-time RT-PCR assay for EV detection [46]. Positive results with cycle threshold values above 40 were classified as negative. Appropriate positive and negative controls were included in all the experiments. Samples were considered as valid when a clear positive or negative result was obtained.

#### Seroprevalence of CMV among pregnant women

Serum collected from the mothers was tested for the presence of anti-CMV immunoglobuline G (IgG) antibodies (AB) using the electrochemiluminescence immunoassay “ECLIA” intended for use on Elecsys and cobas immunoassay analyzers, according to the manufacturer’s instructions. Positive, negative, and cut-off controls were included in all runs, and positive samples were retested to confirm initial positive result.

#### Placental biopsy

A placental biopsy was obtained with a scalpel blade and immediately placed into 10% buffered formalin for transport to the laboratory. Each placental sample was embedded in paraffin wax, sectioned in 5μm tissue pieces and stained with standard haematoxylin–eosin for histopathologic examination. Different histopathological parameters were assessed including a macroscopic description (weight, oedema, haemorrhage, infarct and calcifications) and a microscopic description (inflammation and presence of microorganisms). Additional sections were saved for histochemical staining (PAS, Grocott and Gram) performed in an automated stainer (BenchMark Special Stains- Ventana Roche), following commercial recommendations. An immunohistochemical assay was performed on formaline-fixed paraffin-embedded tissue sections according to standard procedures, using the Ventana-Roche automated immunostainer system (BenchMark XT-Ventana Roche^®^). Antigen retrieval was performed by heat-induced epitope retrieval and B19V (Master Diagnostica^®^, Granada, SP) and CMV (Cell Marque^®^, Rocklin, CA, USA) antibodies were applied according to manufacturing instructions.

#### Infant follow-up

Clinical examination of all newborns, including the evaluation of head circumference and Apgar score at minute 1, 5 and 10, was performed at birth. Dubowitz score for postnatal GA determination was done in live neonates at least 12h after birth[47]. Maternal and child HIV status were registered. All participants were followed using the HMSS in order to check any hospital admissions to MDH during the first six months after birth. No specific imaging or hearing screening was performed in these children.

### Statistical analysis

All data were prospectively collected using standardized questionnaires, which were double entered in specific study databases, created using Openclinica software. Discrepancies were solved after comparison with the original source documents by a senior data clerk, and in close collaboration with the study clinicians. Statistical analyses were performed using Stata 14.1 (Stata Corp., College Station, TX). Study variables were counted and summarized in frequency tables. Univariate and multivariate analyses were performed to identify risk factors for CMV, B19V and EV neonatal infection, separately. Firth logistic regression was used in order to address issues of separability, small sample sizes and bias of the parameter estimates. Variables that were found to be significantly associated with CMV, B19V and EV acquisition in the univariate analysis together with those related at a significance level of *p*<0.10 were entered into a multivariate model. Agreement between the results of DUC RT-PCR assays and those of NPA RT-PCR was assessed through Kappa statistic.

### Ethical considerations

This protocol and all supporting documentation (Informed consent documents, Study questionnaires) were approved by the local bioethics committee of CISM (Comité Institucional de Bioética para Saúde) and by the National Bioethics Committee of Maputo in Mozambique, and by the Ethics Committee of the Hospital Clínic in Barcelona, Spain. Participants were asked to express their willingness to participate in the study by signing (or thumb-printing in case they were illiterate) a consent form. Participation in this study was voluntary, and study-related procedures did not interfere with the pregnant women’s or children’s standard clinical care.

## Results

### Maternal characteristics

**Fig 1:**
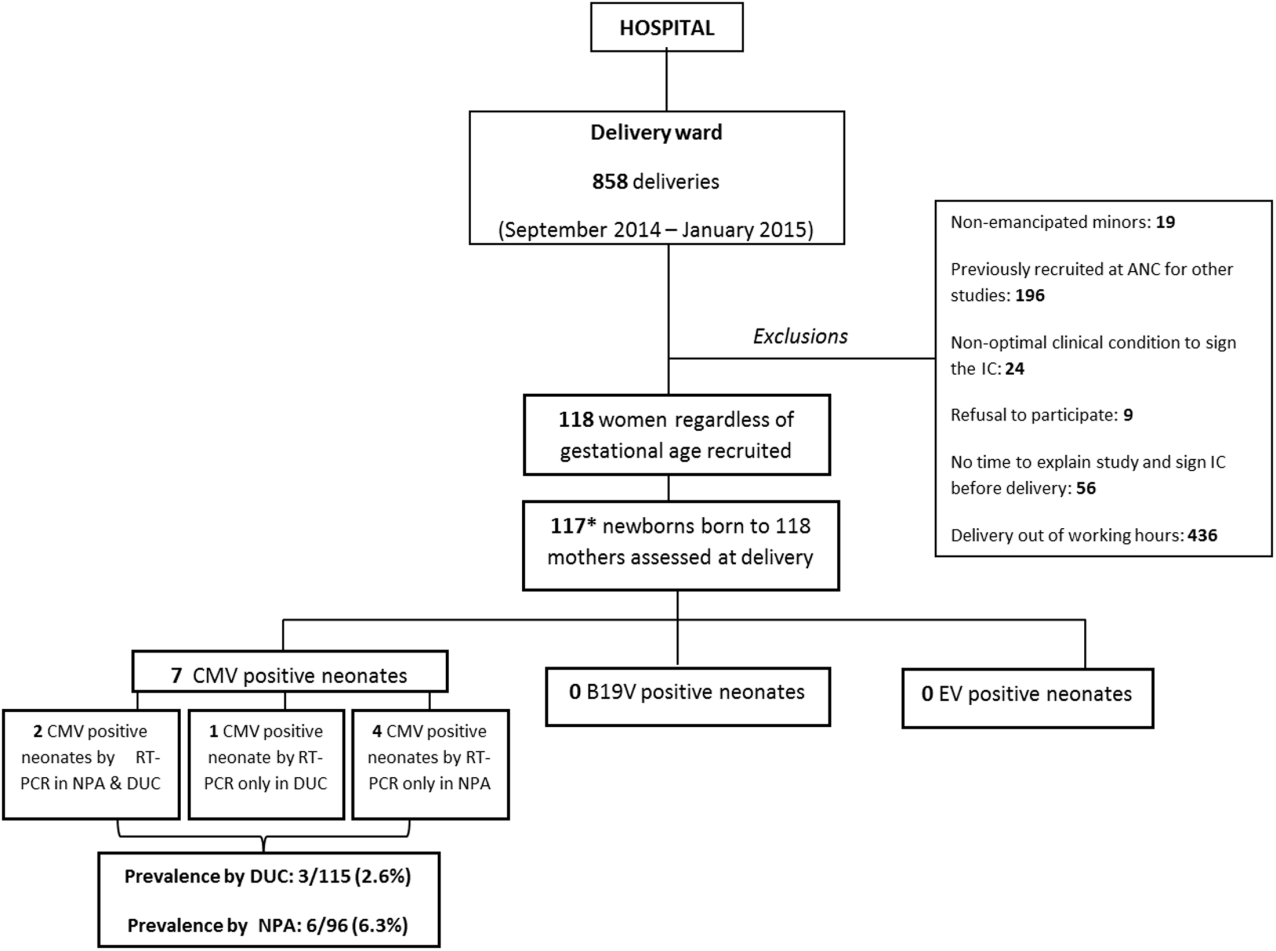
Study profile. ANC: antenatal clinics; IC: informed consent; *One stillbirth whose mother refused to take samples. RT-PCR: real-time polymerase chain reaction, DUC: dried umbilical cord, NPA: nasopharyngeal aspirate.

During the study period, 118 pregnant women were recruited upon delivery at MDH (Fig 1). Table 1 summarizes the socio-demographic and clinical characteristics of participant mothers. Median age of recruited women was 22 years (Interquartile range, IQR 19–29), with >75% being younger than 30 years of age. Nearly a third of women (31.4%, 37/118) were confirmed HIV positive and only one of 118 had a positive syphilis test. Malaria test results were registered as negative in 5/118 women and no information about this disease was available for the rest of mothers. CMV IgG AB were detected in all (100%) women.

**Table 1:** Socio-demographic and clinical characteristics of mothers participating in the study and univariate analysis of maternal risk factors associated to congenital CMV infection measured by different specimens.

	Total mothers recruited, n = 118	Mothers of neonates CMV positive by DUC,n = 3ᶤ	Crude OR (95%CI)ᵟ	p-valueᵟ	Mothers of neonates CMV positive by NPA,n = 6ᶽ	Crude OR (95%CI)ᵟ	p-valueᵟ
**Socio-demographic characteristics**	**n (%)**	**n (%)**			**n (%)**		
**Age in years**				0.61			0.61
< 21	51 (43.2)	2 (66.7)	1.00		4 (66.7)	1.00	
22 to 29	40 (33.9)	0 (0)	0.23 (0.01–4.92)		1 (16.7)	0.23 (0.01–4.92)	
≥30	27 (22.9)	1 (33.3)	1.05 (0.13–8.42)		1 (16.7)	1.05 (0.13–8.42)	
**Seconday or tertiary education**	30 (25.4)	0 (0.0)	0.40 (0.02–8.06)	0.55	3 (50.0)	3.23 (0.68–15.36)	0.14
**Employment**	6 (5.1)	0 (0.0)	2.34 (0.1150.28	0.59	0 (0.0)	1.92 (0.89–41.36)	0.68
**Obstetric History**	**n (%)**	**n (%)**			**n (%)**		
**Age of first pregnancy (median±IQR))**	18.0 (17–20)	18.0 (17–18)	0.91 (0.27–3.11)	0.89	18.0 (18–18)	1.03 (0.76–1.41)	0.84
**Gravidity (mean±SD)**	2.7 (±0.2)	3.7 (±2.2)	1.27 (0.81–2.01)	0.30	1.5 (±0.2)	0.63 (0.31–1.29)	0.21
**Previous abortion n(%)**	11 (9.3)	0 (0.0)	1.26 (0.61–25.97)	0.88	0 (0.0)	0.75 (0.89–14.43)	0.85
**History of current pregnancy**	**n (%)**	**n (%)**			**n (%)**		
**At least 3 antenatal visits during the pregnancy**	69 (58.5)	2 (66.7)	0.78 (0.98–6.15)	0.81	5 (83.3)	1.22 (0.19–8.01)	0.83
**Gestational hypertension**	7 (6.1)	0 (0.0)	2.73 (0.12–62.25)	0.53	1 (16.7)	3.38 (0.47–24.33)	0.23
**Vaginal discharge**	2 (1.7)	0 (0.0)	6.31 (0.25–157.63)	0.26	0 (0.0)	2.72 (0.12–62.86)	0.53
**Investigations**	**n (%)**	**n (%)**			**n (%)**		
**Syphilis positive**	1 (0.5)	0 (0.0)	9.95 (0.34–290.3)	0.32	0 (0.0)	5.12 (0.19–140.92)	0.33
**HIV positive**	37 (31.4)	1 (2.7)	1.26 (0.16–9.89)	0.83	3 (50.0)	2.19 (0.46–10.30)	0.32
**HIV positive in HAART**	33 (89.2)	1 (100.0)	1.25 (0.11–4.43)	0.86	3 (100.0)	2.60 (0.48–14.04)	0.25
**Anemia (<11g/dL)**	55 (72.4)	0 (0.0)	0.13 (0.01–3.26)	0.21	2 (66.7)	0.59 (0.07–4.88)	0.63
**CMV IgG serum antibodies**	118 (100)	3 (100)	0.03 (0.00–1.81)	0.09	6 (100)	0.07 (0.06–3.92)	0.20

IQR (interquartile range). SD: standard deviation. HAART: highly active antiretroviral therapy. CMV: cytomegalovirus. DUC: Dried umbilical cord.

^ᶤ^Results based on 115 valid DUC samples. NPA: Nasopharingeal aspirate.

^ᶽ^Results based on 96 valid NPA samples OR: odds ratio. CI; confindence intervals.

^ᵟ^OR and P-value derived from Firth logistic regression.

**Fig 2:**
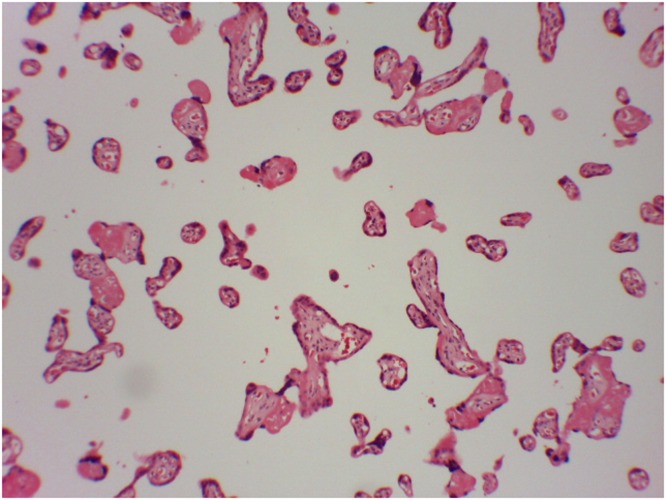
Placental histology from an infant with cytomegalovirus detected in dried umbilical cord blood. Distal villous hypoplasia: many terminal villi are extremely small, with reduced stroma and capillaries number. No inmunohistochemical evidence of CMV could be found.

Distal villous hypoplasia was observed in 27/117 (23.0%) of placental tissue samples evaluated. Neither microorganisms nor other significant findings were detected after evaluating tissue sections with standard tissue staining or in the immunohistochemical studies (Fig 2). No maternal risk factors associated to neonatal cCMV were found in the univariate analysis and thus, multivariate analysis was not performed (Table 1).

### Neonatal outcomes and infant follow-up

One hundred and eighteen delivery outcomes from the 118 pregnant women were recorded (100%) at MDH. Characteristics of neonates born of mothers participating in the study are shown in Table 2. Neonatal outcomes included 110 live term babies, 7 preterm and 1 case of stillbirth born at 41 weeks of GA, whose mother refused permission to sample the foetus. Twenty-one (17.8%) newborns had low-birth-weight and three of them (2.5%) presented microcephaly at birth[48].

**Table 2:** Clinical characteristics of neonates born to mothers participating in the study.

	Neonates at birth n = 118	Neonates CMV positive by DUC n = 3ᶧ	Crude OR (95%CI)ᵟ	p-valueᵟ	Neonates CMV positive by NPA n = 6ᶽ	Crude OR (95%CI)ᵟ	p-valueᵟ
**Clinical characteristics of newborns**ᶤ	**n (%)**	**n (%)**			**n (%)**		
**Gestational age at birth (live birth)**				0.65			0.21
Term newborn	110 (94.1)	3 (100.0)	1.00		5 (83.3)	1.00	
Pre term newborn	7 (5.9)	0 (0)	2.01 (0.95–42.62)		1 (16.7)	3.54 (0.49–25.53)	
**Stillbirth**	1 (0.9)	0 (0)	_	_	0 (0)	_	_
**Low birth weight (<2500gr)**	21 (17.8)	0 (0)	0.61 (0.03–12.21)	0.75	1 (16.7)	1.23 (0.19–8.09)	0.22
**Head circumference in cm (mean±SD)**	35.6 (0.4)	39.7 (5.2)	1.13 (0.99–1.29)	0.08	36.2 (2.9)	2.69 (0.12–62.15)	0.54
**Microcephaly**	3 (2.5)	(0)	4.43 (0.19–103.20)	0.35	1 (16.7)	9.65 (1.07–87.12)	**0.04**
**Perinatal asphyxia**	2 (1.7)	0 (0)	10.62 (0.36–309.66)	0.17	0 (0)	2.69 (0.12–62.15)	0.54
**Jaundice**	0 (0)	0 (0)	_	_	0 (0)	_	_
**Purpura**	0 (0)	0 (0)	_	_	0 (0)	_	_
**Dubowitz neurological score (mean±SD)**ˠ	30.8 (0.2)	31.0 (0.8)	0.88 (0.64–1.22)	0.46	31.7 (1.02)	1.14 (0.79–1.65)	0.47
**Malformations at birth**	2 (1.7)	0 (0)	6.25 (0.25–156.21)	0.26	0 (0)	2.69 (0.12–62.15)	0.54
**Sick at birth**	2 (1.7)	0 (0)	6.25 (0.25–156.21)	0.26	0 (0)	2.69 (0.12–62.15)	0.54
**Outcome**							
**Admitted first 6 months of life**	7 (5.9)	0 (0)	2.01 (0.95–42.62)	0.65	1 (16.7)	3.55 (0.49–25.52)	0.21
**Death after birth**ᶬ	2 (1.7)	0 (0)	6.25 (0.25–156.21)	0.26	0 (0)	2.69 (0.12–62.15)	0.54

CMV: cytomegalovirus. DUC: Dried umbilical cord.

^ᶧ^Results based on 115 valid DUC samples. OR: odds ratio. CI; confidence intervals.

^ᵟ^OR and P-value derived from Firth logistic. NPA: Nasopharingeal aspirate.

^ᶽ^Results based on 96 valid NPA samples.

^ᶤ^Results based on 117 patients. Mother of a stillbirth refused to take sample of the baby.

^ˠ^Suboptimal neurological score following Dubowitz: <30.5.

^ᶬ^ Death (including stillbirth and any deaths in the first 6 months after birth)

At least one specimen was collected from 117 neonates. A hundred and fifteen of the 117 DUC samples obtained were valid for viral determination. All of them were negative for B19V and EV.

Three of the 115 (2.6%) valid DUC samples were positive for CMV. This virus was also detected in 6/96 (6.3%) valid NPA samples obtained. A total of 7/117 (6.0%) newborns tested had at least one positive sample for CMV. Prevalence of cCMV infection measured by DUC among HIV-exposed neonates was 2.7% (1/37) and 8.1% (3/37) when CMV was detected through NPA, although no significant difference was found compared to the prevalence among HIV-unexposed in both cases (Table 1). One of the NPA-CMV positive cases was born preterm and with low birth weight while all neonates DUC-CMV positive were healthy at birth. CMV infection was associated to a higher risk of microcephaly at birth when CMV was determined by NPA (OR 9.65, 95% CI 1.07–87.12, p = 0.04) in the univariate analysis (Table 2).

Through HMSS, 7/117 infants born to mothers participating in the study were detected as admissions at MDH at least once during their first 6 months of life (Table 2). Two of them admitted within the first 24h after birth died due to clinical sepsis. Other causes of admission included perinatal asphyxia, bronchiolitis, diarrhoea and malaria. Among neonates were CMV was detected, only the preterm baby with a positive NPA for CMV was admitted at birth. In all of these cases, children were discharged fully recovered. No additional follow-up investigations were conducted.

#### Comparison of dried umbilical cord and nasopharyngeal aspirate RT-PCR Assays for CMV determination

A total of 94 pairs of samples (DUC and NPA) from 94 neonates were available for testing CMV by RT-PCR assay and comparing results. Of these, 2/94 neonates (2.1%) were positive for CMV by any test. A newborn with a positive RT-PCR assay through DUC had a negative NPA result, and the NPA RT-PCR assay also identified four additional neonates as infected although their DUC RT-PCR were negative (Fig 1). The overall concordance of the RT-PCR assay through DUC with their correspondent through NPA was moderate (Kappa = 0.42 and p<0.001).

## Discussion

This study is a first attempt at proactively investigating vertical transmission of CMV, B19V and EV in Mozambique. The study was an opportunistic attempt to explore congenital transmission of these viruses, and was not specifically designed to evaluate the performance of the RT-PCR method of identification of cCMV in neonates as compared with the “gold-standard” for the detection of CMV (isolation of the virus in rapid cultures or PCR assay) since that has already been demonstrated[49–51]. The study shows a prevalence of cCMV infection assessed through dried umbilical cord samples of 2.6% and through NPA of 6.3%. Although specimens utilized in this study have not been validated for their use in cCMV diagnosis, they highlight a high burden of vertical CMV transmission. Contrarily, B19V and EV congenital transmission in the newborns was not found in this cohort although only DUC samples were analyzed. The prevalence of cCMV in this study detected through DUC was likely underestimated, as it has been demonstrated that real-time dried-blood-spot PCR assay has a lower sensitivity compared with the standard saliva rapid culture[26]. The dried umbilical cord samples have previously been used for retrospective studies to diagnose cCMV[29, 30] and although this sample type has never been compared to other accepted specimens for cCMV diagnosis, it would however be reasonable to assume that a positive DUC sample is likely a true positive. On the other hand, NPA has never been previously assessed as a specimen valid for cCMV diagnosis. A significant limitation of this study includes the fact that up to 30% of CMV-seropositive women will secrete CMV in their vaginal fluid, something that could possibly contaminate with CMV the nasopharynx of neonates[52]. Thus, the prevalence of 6.3% found in this study through NPA may potentially overestimate the true prevalence.

If the cCMV prevalence found through DUC can be a proxy of the true prevalence, these findings suggest higher prevalence rates than those reported in newborns from industrialized countries (<1%)[14] and falls within the range reported in a systematic review for developing countries (0.6%–6.1%)[53]. However, that review excluded studies reporting data from high at-risk populations for CMV transmission, such as HIV infected mothers, and restricted inclusion to studies having used the recommended gold standard specimens (saliva and urine) and techniques (cultures and PCR)[54]. Although it is likely that our cCMV prevalence was underestimated for the aforementioned reasons, other studies conducted in HIV endemic areas have reported similar congenital CMV prevalence to those shown here. A study conducted in Nigeria found a rate of 3.8% among neonates born to mothers with a low prevalence of HIV (4.8%)[55]. High prevalence of cCMV in HIV-exposed infants has been previously reported in two settings (South Africa and Zambia) with a maternal HIV prevalence similar to the one documented in our study area[23, 56–58]. The South African study was conducted among 748 HIV-exposed infants found a cCMV prevalence of 2.9%. No comparison with HIV-unexposed was performed [23]. Overall prevalence of cCMV among high-risk newborns admitted to a referral neonatal unit in Zambia was 3.8%, and 11.4% in those infants exposed to maternal HIV (Adjusted OR 6.66, 95% CI 2.13–20.9)[58]. HIV prevalence in our maternal cohort was very high (31.4%) and almost 90% of mothers were under highly active antiretroviral therapy (HAART), possibly explaining why the prevalence of cCMV was not higher among HIV-exposed newborns and why differences between HIV-exposed and unexposed neonates (2.7% *vs*. 2.6%%, OR 1.26 95% CI 0.16–9.89, p = 0.83) were not found[59]. Reasons to explain the difference between Zambian study results and our findings could be that the Zambian study was performed on admitted and therefore sick neonates and our study was conducted at time of birth. Another reason may be a better immune status of our HIV-infected mothers although information about HAART in this Zambian study was not available[58]. Immunosuppression in HIV-infected pregnant women likely leads to increased incidence of reinfection or reactivation, or prolonged CMV viral shedding, lengthening the opportunity for congenital transmission[21, 60]. Moreover, an association of CMV transmission with advanced maternal immunosuppression has been previously described[23].

No risk factors independently associated with cCMV were found. Primiparity, acute placental malaria, HIV-exposure and jaundice have all been reported as independent risk factors for cCMV infection by other authors[19, 28, 58, 61]. Caution is needed when interpreting our findings, since sample size was small and likely insufficient to detect significant differences among infected and uninfected neonates. It has been estimated that 90% of infected newborns do not have obvious clinical signs of CMV congenital infection and of them, only 15% will develop long-term neurological sequelae, especially, neurosensory hearing loss[14]. However, no further examinations and follow-up were performed beyond birth and burden of hearing loss was not explored. cCMV infection is an important cause of hearing loss and better strategies to detect children at risk of this complication in LIC should be developed.

Maternal CMV IgG seroprevalence in this study was 100%. Immune status of mothers in our cohort prior to pregnancy was unknown. In these cases, isolated detection of CMV IgG or detection of specific IgM AB are inadequate single measures to diagnose maternal primary infection[28]. Estimates suggest that around 75% of all cCMV cases in industrialized countries occur in babies born to women with non-primary maternal infection (those women who are CMV seropositive before pregnancy[28, 62–64]) and the risk of intrauterine transmission has been estimated at 1% in CMV-seropositive mothers[14]. IgG CMV seroprevalence in developing countries is generally over 90% by adolescence and over 95% by early adulthood[53] and it has been demonstrated that the incidence of cCMV infection is parallel to maternal seroprevalence[28], suggesting that most of cases of vertical transmission of this virus result from non-primary maternal infection and may be due to reactivation of latent virus or reinfection with a new cytomegalovirus strain[28, 62–64]. This is the reason behind the current recommendations issued by The International Congenital Cytomegalovirus Recommendations Group of not conducting universal serological screening of pregnant women for primary CMV infection[28].

Maternal infections with B19V, CMV and EV have been associated with intrauterine foetal death[65]. This study did not focus in cases of abortion and only one stillbirth was registered during recruitment, which hinders our capacity to associate them to the aforementioned pathogens.

Our findings suggest a low prevalence of B19V and EV infections in Southern Mozambique. No B19V congenital infection in newborns was found in this study. To our knowledge, no prospective screening of congenital B19V and EV infections among neonates in developing countries has been conducted to date. A South African study exploring prevalence of B19V infection among pregnant women found 20 asymptomatic neonates born to IgM positive mothers, although no samples from newborns were obtained[66]. Different African studies on maternal B19 V seroprevalence found IgG AB between 24.9 and 80% and IgM between 3 and 19%[66–70]. Unfortunately, maternal seroprevalence was not performed in this study and further research should be done in order to know the real burden of congenital B19V.

Similarly, all babies were negative for EV and maternal seroprevalence studies were not performed. Data on incidence and consequences of EV during pregnancy and clinical outcomes are globally scarce. Case reports and small case series have suggested that EV infection may cause foetal loss, and maternal infections during the 2^nd^ and 3^rd^ trimester may also lead to in utero foetal anomalies and death, but also to severe neonatal infections[37]. However, no prospective studies investigating EV maternal prevalence and risk of transmission have been conducted.

The few and unspecific anatomopathological findings documented in this study, consisting on an accelerated placental maturation, could have resulted from a variety of causes, including maternal preeclampsia or other states of maternal vascular underperfusion, or, more likely in our setting, due to malnutrition or infectious such as HIV or malaria. However, the lack of ovular membranes and umbilical cord in placental samples did not allow ruling out possible infections of these tissues.

Our study has several limitations. First, diagnosis of cCMV through DUC specimen is a methodology not recommended for neonatal CMV screening, particularly as urine and saliva have been demonstrated to be the most reliable specimens [26, 28]. The use of NPA for cCMV has not been validated and this specimen could be contaminated by maternal secretions contained CMV, leading to an overestimation of the true cCMV prevalence. However, a similar issue occurs with saliva samples, since the risk of contamination of saliva samples with breast milk exists. We however chose to use RT-PCR methods, with known good performance, in those samples available from the study, both because the kind of samples and the molecular screening techniques could be a good approach to study several viruses simultaneously. Further studies to validate these particular specimens for cCMV diagnosis would help to know their specificity and positive predictive value, and shed a light on why an important number of samples provided invalid results. Second, further follow-up and additional investigations beyond six months of life were not performed and hearing impairment, which is frequently progressive and usually develops later during infancy, was not measured, leading to a potential underestimation of CMV- associated morbidity. Third, the study lacked sufficient statistical power to detect independent risk factors associated to higher risk of congenital CMV, B19V and EV infections given the small sample size, and the few positive results. Additionally, it is known that both, B19V and EV show seasonal or even epidemic patterns [71, 72]. Considering that the epidemiology of these viruses in unknown in Mozambique, we may have failed, during the short study period, to capture natural transmission of the pathogens. Finally, B19V and EV were only assessed in dried umbilical cord samples at birth and maternal seroprevalence of these viruses or their presence in other fluids such as amniotic fluid was not investigated. Understanding also that these viruses may not be detected in cord blood in the absence of a true viraemia, it appears difficult to properly correlate the burden of maternal infection and the associated vertical transmission of these viruses. Thus, the prevalence observed in this study could be importantly underestimated.

In conclusion, despite the small sample size and the use of non-standard specimens, this study demonstrates that the prevalence of vertical transmission of CMV may be high in southern Mozambique, although further research is needed to assess its clinical relevance in this area. Studies validating the sensitivity and specificity of NPA for CMV screening would be required before considering it for clinical use. Congenital B19V and EV infections seem to be less prevalent in this area. Further research to evaluate the consequences of vertical transmission of these viral infections in resource-constrained settings is needed.
